# Extracranial and Intracranial Vasculopathy With “Moyamoya Phenomenon” in Association With Alagille Syndrome

**DOI:** 10.3389/fneur.2018.01194

**Published:** 2019-01-29

**Authors:** Siobhan Delaney, Ged O'Connor, William Reardon, Stephen J. X. Murphy, Sean Tierney, Barbara M. Ryan, Holly Delaney, Colin P. Doherty, Michael Guiney, Paul Brennan, W. Oliver Tobin, Dominick J. H. McCabe

**Affiliations:** ^1^Department of Neurology, The Adelaide and Meath Hospital, Dublin, Incorporating The National Children's Hospital (AMNCH)/Tallaght University Hospital, Dublin, Ireland; ^2^Stroke Service, AMNCH/Tallaght University Hospital, Dublin, Ireland; ^3^Department of Neurology, University Hospital Waterford, Waterford, Ireland; ^4^National Centre for Medical Genetics, Our Lady's Children's Hospital, Dublin, Ireland; ^5^Vascular Neurology Research Foundation, C/O Department of Neurology, AMNCH/Tallaght University Hospital, Dublin, Ireland; ^6^Department of Vascular Surgery, AMNCH/Tallaght University Hospital, Dublin, Ireland; ^7^Department of Gastroenterology, AMNCH/Tallaght University Hospital, and Department of Clinical Medicine, Trinity College Dublin, Dublin, Ireland; ^8^Department of Radiology, AMNCH/Tallaght University Hospital, Dublin, Ireland; ^9^Department of Neurology, St James's Hospital, Dublin, Ireland; ^10^Department of Radiology, St James's Hospital, Dublin, Ireland; ^11^Department of Neuroradiology, Beaumont Hospital, Dublin, Ireland; ^12^Department of Neurology, College of Medicine, Mayo Clinic, Rochester, MN, United States; ^13^Department of Clinical Neurosciences, Royal Free Campus, UCL Institute of Neurology, London, United Kingdom; ^14^Irish Centre for Vascular Biology, Dublin, Ireland; ^15^Academic Unit of Neurology, School of Medicine, Trinity College Dublin, Dublin, Ireland

**Keywords:** Alagille syndrome (AGS), internal carotid artery (ICA), moyamoya phenomenon, JAG1 gene, transient ischaemic attack

## Abstract

**Background:** Alagille syndrome (AGS) is an autosomal-dominant, multisystem disorder caused by mutations in the JAG1 gene.

**Case Description:** A 34-year-old man was referred to our service 10 years ago with focal seizures with impaired awareness and transient slurred speech. He had a 5-year history of intermittent left monocular low-flow retinopathy. He has a family history of AGS. General examination revealed mild hypertension, aortic regurgitation, and livedo reticularis. Neurological examination was normal.

**Investigations:** He had mild hyperlipidaemia and persistently-positive lupus anticoagulant consistent with primary anti-phospholipid syndrome. Color Doppler ultrasound revealed low velocity flow in a narrowed extracranial left internal carotid artery (ICA). MR and CT angiography revealed a diffusely narrowed extracranial and intracranial left ICA. Formal cerebral angiography confirmed severe left ICA narrowing consistent with a left ICA “vasculopathy” and moyamoya phenomenon. Transthoracic echocardiogram revealed a bicuspid aortic valve and aortic incompetence. Molecular genetic analysis identified a missense mutation (A211P) in exon 4 of the JAG1 gene, consistent with AGS.

**Discussion:** AGS should be considered in young adults with TIAs/stroke and unexplained extracranial or intracranial vascular abnormalities, and/or moyamoya phenomenon, even in the absence of other typical phenotypic features. Gene panels should include JAG1 gene testing in similar patients.

## Introduction

Alagille syndrome (arterio-hepatic dysplasia) is an autosomal-dominant, multisystem disorder with a prevalence of 1 in 70,000 (1, 2), typically characterized by dysmorphic facial features, posterior embryotoxon, axial skeletal defects, peripheral pulmonary arterial hypoplasia, and intrahepatic cholestasis (3). There is a dearth of interlobular bile ducts, aberrant angiogenesis, and vascular morphogenesis (2), with renal dysfunction, pancreatic insufficiency, growth, and intracranial abnormalities (3). Moyamoya phenomenon is a rare intracerebral vasculopathy, mainly due to stenosis of the distal internal carotid, proximal middle, and anterior cerebral arteries, with associated collateral vessel formation (4). Moyamoya phenomenon has rarely been described in Alagille syndrome (AGS) (4–8).

## Case Description

This 34 year old left-handed man was referred to our service 10 years ago following an episode of intermittent disorientation, altered awareness, patchy global amnesia, transient slurred speech, and tremulousness lasting <24 h, attributed to likely focal seizures with impaired awareness. He had experienced intermittent, stereotyped left monocular visual blurring with definite left monocular vertical oscillopsia over the preceding 5 years; episodes occurred 1–3 times/month, lasted 20–30 min and resolved over 10 min with eye closure, followed by severe left temporal aching headaches and left monocular photophobia within 5 s of initial symptom onset which then lasted for the same duration as the visual symptoms. There were no other features of migraine or trigeminal autonomic cephalgia. He also experienced independent, identical, left monocular blurring without oscillopsia or headache on exposure to sunlight, resolving within 5 s when he stopped looking at the light and closed his eyes, consistent with left monocular TIAs due to low-flow retinopathy. He initially had untreated hypertension for 2 years, a 4 pack-year smoking history and consumed 35 units of alcohol/week, with no other relevant history. Paternal grandfather died suddenly at age 41 years (unknown cause). Mother has Parkinson disease since age 55. His father, who died from suspected oropharyngeal cancer, was an obligate carrier of the AGS mutation by his position in the pedigree (see below). A paternal aunt has AGS and required pacemaker insertion for “cardiac reasons”; another paternal aunt had a stroke at age 40, but “AGS status” has not been clarified. One brother has genetically-confirmed AGS without apparent clinical manifestations. Three paternal first cousins have confirmed AGS with clinically supportive findings.

General examination revealed hypertension (168/98 mmHg), livedo reticularis, a slightly pointed chin, aortic regurgitation, and diffuse abdominal tenderness without guarding, rigidity or audible abdominal bruits. Cognitive testing revealed decreased verbal fluency (seven “F” words in 1 min), but was otherwise normal. Neurological examination of the cranial nerves, fundi, and limbs was normal.

Fasting total cholesterol was 4.2 mmol/L, with LDL cholesterol 2.7 mmol/L and normal triglycerides before commencing statins. Otherwise, routine hematological and biochemical screening was normal. ANA, ANCA, ENA, rheumatoid factor, anti-cardiolipin and anti-beta 2 glycoprotein I antibodies were negative. Lupus anticoagulant was persistently positive consistent with primary anti-phospholipid syndrome. Electrocardiogram revealed left ventricular hypertrophy. Transthoracic echocardiogram revealed a bicuspid aortic valve, moderate aortic incompetence and symmetrical left ventricular hypertrophy. EEG did not show any epileptiform changes. Color Doppler ultrasound demonstrated low flow in a narrowed left internal carotid artery (LICA), with a LICA peak systolic velocity of 25 cm/sec, but was otherwise normal. Serial brain MRIs revealed normal brain parenchyma over a 10-year period, but features suspicious of moyamoya phenomenon.

**Figure 1:**
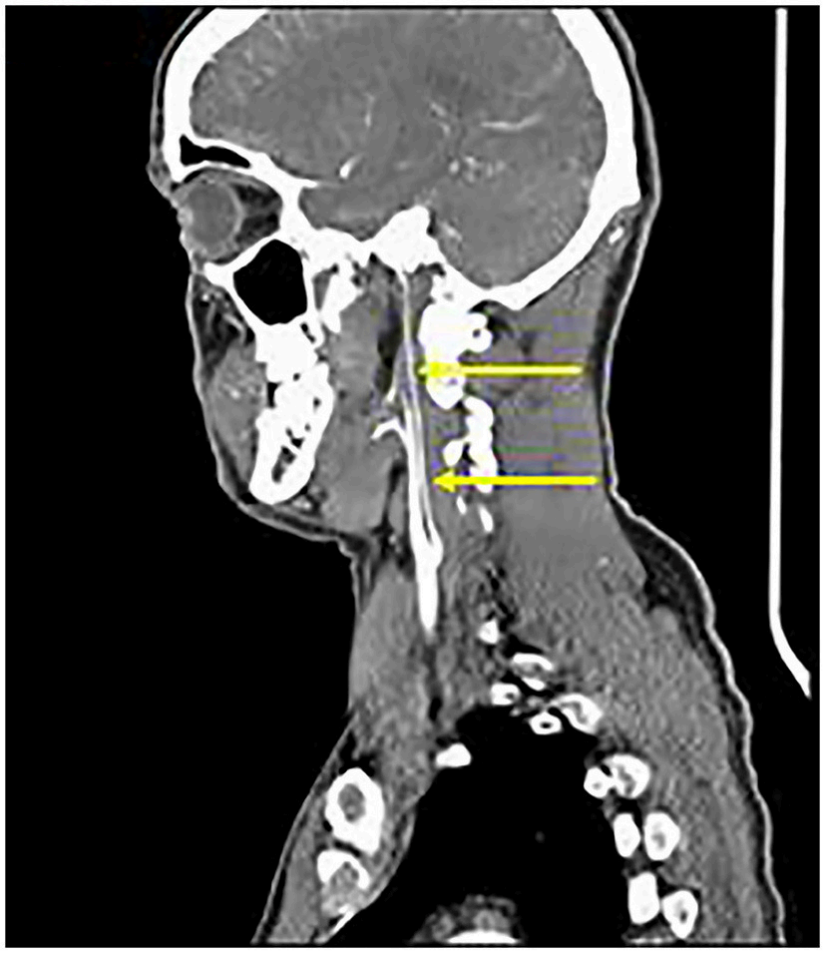
Extracranial CTA: Diffuse severe left ICA narrowing from 0.5 cm beyond the carotid bifurcation (arrows), consistent with “Alagille vasculopathy.”

**Figure 2:**
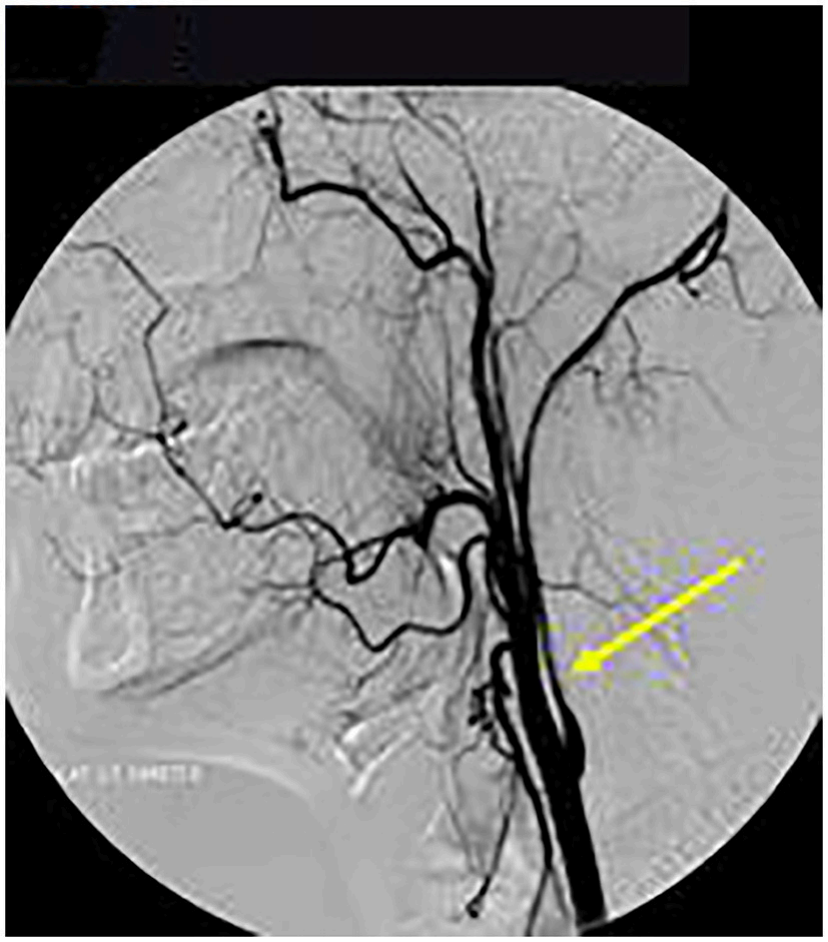
Formal Catheter Angiography: *Sagittal views* following left carotid injection showed ≥75% extracranial LICA stenosis (arrow). LICA was occluded in the supraclinoid segment after the left ophthalmic artery origin with moyamoya phenomenon (not shown).

**Figure 3:**
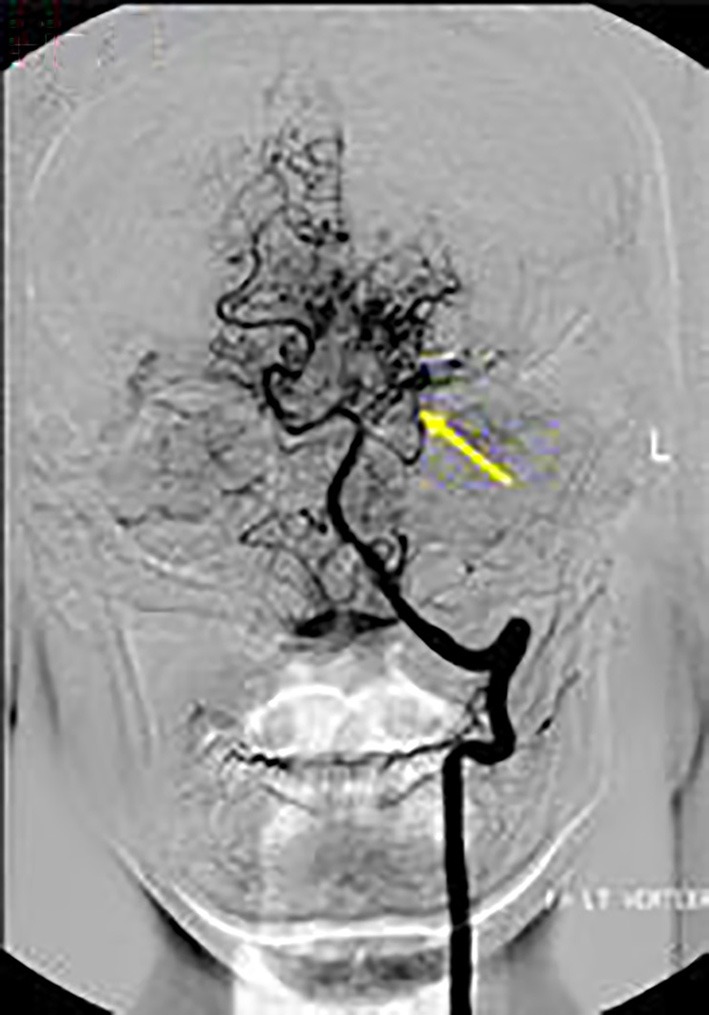
*AP views* following left vertebral artery injection showed moyamoya phenomenon around the proximal left PCA (arrow).

Axial T1 fat-saturated MRI neck vessels revealed a diffusely thickened wall of the left CCA (not shown). Extracranial MRA (not shown) and CTA (Figure 1) revealed a diffusely-narrowed left ICA. Formal cerebral angiography confirmed severe left ICA narrowing suspicious of either prior left ICA dissection or vasculopathy (Figure 2), and confirmed moyamoya phenomenon in the anterior and posterior circulations (Figure 3). The proximal right middle cerebral artery also showed features of moyamoya phenomenon (not shown). Pre- and post-acetazolamide SPECT imaging revealed exhausted haemodynamic reserve in the left ICA territory.

Molecular genetic analysis revealed heterozygosity for a missense G>C mutation in exon 4 of the JAG1 gene on chromosome 20 resulting in substitution of proline for alanine at codon 211 (p.Ala211Pro). Pathogenicity was confirmed by familial segregation studies.

## Clinical Course

He was treated with aspirin 75 mg daily and dipyridamole MR 200 mg twice daily with initial resolution of left monocular visual symptoms. He represented 5 months later with a 2-week history of intermittent left temporal nagging headaches, followed by sudden-onset left monocular visual blurring lasting 2 min with sudden resolution, but no associated oscillopsia or focal neurological features. These symptoms only occurred whilst sitting upright. He had no recurrent visual blurring in response to bright lights or other hemodynamic ischaemic triggers, no recent neck trauma or independent headaches. Repeat MRI brain showed no new ischaemia. Extracranial and intracranial vessels were unchanged on repeat MRA and CTA, respectively. A clinical diagnosis of recurrent left monocular TIAs, likely due to low-flow retinopathy and haemodynamic ischaemia was made, although embolic phenomena could not be completely excluded. His antiplatelet regimen was changed to clopidogrel 75 mg daily. He had persistent gastro-intestinal intolerance of clopidogrel, and subsequently of enteric-coated aspirin 150 mg daily in combination with dipyridamole MR 200 mg daily or BD, ultimately necessitating reversion to 150 mg of aspirin monotherapy daily. Anticoagulation with warfarin was avoided due to the risk of intracranial hemorrhage with moyamoya phenomenon. He experienced intermittent migraine with and without aura, and migraine aura sine cephalgia over the following 15 months which then settled. Repeat formal 6-vessel catheter angiography showed slightly improved cross-flow from the right anterior cerebral into the left anterior and middle cerebral arteries following right carotid injection, but was otherwise unchanged compared with prior angiography 2 years earlier.

When last assessed, he had experienced no further TIA or stroke over the following 9.5 years. He had intermittent abdominal pain and vomiting exacerbated by eating, attributed to mesenteric ischaemia, with reduced oral intake and weight loss. He was taking aspirin 150 mg daily, atorvastatin 10 mg nocte, amlodipine 10 mg daily, esomeprazole 40 mg daily, hyoscine butylbromide 10 mg BD, and paracetamol 1 g QDS PRN. He continued smoking 10 cigarettes daily, but successfully stopped drinking alcohol. Neurological exam was unchanged.

He had no new changes on serial brain MRIs or extracranial and intracranial CTAs. Abdominal CTA revealed occlusion of the coeliac axis origin with distal reconstitution from multiple collaterals, 50% superior mesenteric artery stenosis with post-stenotic dilation, and mild right renal artery stenosis 1 cm from its origin, necessitating ongoing follow-up with gastroenterology and vascular surgical colleagues.

## Discussion

AGS is caused by mutations in JAG1 encoding the Jagged-1 protein (>95%), a ligand for the Notch receptor family, or by mutations in the NOTCH2 gene itself (<5%) (9, 10). JAG1 loss-of-function deletions, frameshift or nonsense mutations are identified in ~70% of patients with AGS; missense mutations represent 12% (11) and compromise Jagged-1's role in modulating vascular development (12). The Notch inter-signaling pathway is involved in several developmental microsystems, explaining the phenotypic diversity of AGS (9). Over 230 causative mutations have been described in AGS (13).

Several features in this case enhance our understanding of the AGS phenotype. Ocular involvement is well-described, including posterior embryotoxon, fundal hypopigmentation, optic disc abnormalities and iris abnormalities (14). The stereotyped episodes of visual blurring and monocular oscillopsia were consistent with left ocular TIAs due to ipsilateral low-flow retinopathy associated with left CCA and severe LICA vasculopathy with AGS. It is reassuring that his cerebrovascular symptoms were controlled during medium-term follow up with active treatment of vascular risk factors and aspirin monotherapy, despite ongoing smoking. Despite persistently-positive lupus anticoagulant assays consistent with primary anti-phospholipid syndrome, we did not treat him with warfarin due to clear evidence of a risk of hemorrhage with anticoagulation in patients with moyamoya phenomenon (15). Surgical revascularization may be effective for those with progressive moyamoya phenomena, affording a 96% probability of being stroke-free 5 years post-operatively (16). However, in one pediatric series of surgical revascularization for moyamoya phenomenon in five patients with AGS, 2 (40%) had post-op complications: one patient had a peri-operative stroke and one had a fatal thalamic hemorrhage 2 years after surgery (17). Surgical revascularization has not been indicated in our case because his cerebrovascular symptoms have been extremely well controlled with stable, serial neuroimaging on medium-dose aspirin monotherapy to date.

Although uncommon, cerebral vasculopathy is described in AGS, including aneurysms, dolichoectasia and moyamoya phenomenon (18); “vascular anomalies” and stroke affect 10-14% of patients (6, 19). Close clinical and neuroradiological follow-up of this case illustrates that moyamoya phenomenon may occur in an adult with AGS and may remain stable over 10 years. Other well-described neuroradiological findings which may be seen in an overall population of patients with moyamoya phenomenon include the “ivy sign” (due to slow flow in dilated leptomeningeal collateral vessels on FLAIR MRI) and the “brush sign” (due to prominence of deep medullary veins draining into subependymal veins on susceptibility-weighted MRI) which may be indicative of disease severity (20). The ivy sign was not present in our patient, but as stated above, pre- and post-acetazolamide SPECT imaging revealed exhausted haemodynamic reserve in the left ICA territory. T2^*^ (gradient-echo MRI) rather than susceptibility-weighted MRI was performed during serial MRIs at our hospital to look for microhemorrhages, so we could not comment on the presence or absence of a “positive brush sign” in this case.

The etiology of the intermittent focal seizures/possible complex partial status epilepticus with an amnestic state could be independent of AGS, and may have been triggered by alcohol in an individual susceptible to developing seizures. It is possible he had haemodynamic TIAs due to transient focal cerebral ischaemia in the setting of exhausted haemodynamic reserve associated with AGS-vasculopathy and moyamoya phenomenon; this could have provoked secondary seizure activity which did not recur on aspirin and other secondary preventive medications despite intermittently drinking alcohol for the next 5 years. Although he did not have any other classical, definitive phenotypic features of AGS, he shares the pathogenic family mutation which also caused the vasculopathy affecting the coeliac axis origin and superior mesenteric artery with severe ischaemic abdominal pain that was not responsive to aspirin. This emphasizes the importance of asking about ischaemic symptoms in other vascular beds, including recurrent abdominal pain after eating suggestive of mesenteric ischaemia, and of taking a detailed extended family history which would prompt screening for AGS in similar cases.

Moyamoya syndrome is the term used to describe moyamoya phenomenon which occurs in association with a number of other established genetic disorders, including e.g., sickle cell disease, neurofibromatosis type I and Down syndrome and Turner syndrome (16, 21). More recently, several RNF213 gene mutations have been described in patients with moyamoya disease (22)^1^, with R4810K mutations associated with ischaemic moyamoya and A4399T mutations predominantly associated with hemorrhagic moyamoya phenomena, respectively (23). Functional studies assessing the potential effect of this specific heterozygous JAG1 missense G>C mutation on angiogenesis *in vivo* have not been possible. However, prior experimental studies have suggested that over-expression of Jagged-1 could promote angiogenesis and neovascularization (24). Those functional data which do exist, relating to a small subset of the total number of missense mutations in the JAG1 gene, show that these mutations generally mediate their clinical impact through haplo-insufficiency, consequent on altered processing of the newly translated protein, which is therefore not targeted to the cell surface (11, 25).

In conclusion, AGS should be considered in young adults with TIAs in association with unexplained extracranial or intracranial vascular abnormalities, and/or moyamoya phenomenon, even in the absence of other typical clinical syndromic features of AGS. With clear, evolving evidence of an association between moyamoya phenomenon and genetically-confirmed AGS, it is reasonable to use the term “moyamoya syndrome” if moyamoya phenomenon is identified in adult patients with AGS. Clinicians should ensure that gene panels include JAG1 gene testing in similar patients, and offer advice regarding treatment of vascular risk factors and secondary stroke prevention.

## Ethics Statement

The patient gave written and informed consent in accordance with and utilizing the British Medical Journal template for obtaining patient consent for publications.

## Author Contributions

SD was involved in data collection, data interpretation, and drafting the manuscript. DM was involved in the concept and design of the report, data collection over 10 years, and critically appraised the manuscript for important intellectual content and supervised the report. WR was involved in data interpretation, data collection, molecular genetic screening, and critical revision of the manuscript. All other authors were involved in data collection and critically appraised the manuscript for important intellectual content.

### Conflict of Interest Statement

The authors declare that the research was conducted in the absence of any commercial or financial relationships that could be construed as a potential conflict of interest.

## Footnotes

^1^RNF213 gene (ring finger protein 213). Genetics Home Reference: Your guide to understanding genetic conditions. Lister Hill National Center for Biomedical Communications, U.S. National Library of Medicine, National Institutes of Health, Department of Health & Human Services. 2019: 1–4. Online data and printable PDF available from: https://ghr.nlm.nih.gov/gene/RNF213.
